# Normal reference ranges for urinary δ‐aminolevulinic acid and porphobilinogen levels

**DOI:** 10.1002/jmd2.12173

**Published:** 2020-10-01

**Authors:** Sagar Agarwal, Bahru Habtemarium, Yuanxin Xu, Amy R. Simon, Jae B. Kim, Gabriel J. Robbie

**Affiliations:** ^1^ Alnylam Pharmaceuticals Cambridge Massachusetts USA

**Keywords:** acute hepatic porphyria (AHP), acute intermittent porphyria (AIP), ALN‐AS1, aminolevulinate synthase 1 (ALAS1), givosiran, porphobilinogen (PBG), small interfering ribonucleic acid (siRNA), upper limit of normal (ULN), δ‐aminolevulinic acid (ALA)

## Abstract

Acute hepatic porphyria (AHP) is a family of rare, serious, and potentially life‐threatening metabolic disorders caused by mutations in genes encoding enzymes involved in hepatic heme biosynthesis. AHP is characterized by accumulation of neurotoxic heme intermediates, δ‐aminolevulinic acid (ALA), and porphobilinogen (PBG), which are thought to be causal for the disease manifestations. Novel therapeutic treatments such as givosiran, an RNA interference therapeutic that was recently approved for treatment of adults with AHP, are focused on reducing the levels of ALA and PBG in patients toward levels observed in a healthy population. While there are two published reports on the distribution of urinary ALA and PBG levels in healthy subjects, these lacked the required details to enable the calculation of reference limits for ALA and PBG. Therefore, urinary ALA and PBG levels were quantified in 150 healthy subjects using a validated liquid chromatography tandem mass spectrometry (LC‐MS/MS) method that is highly sensitive, specific, accurate, and reproducible. These data were used to establish the upper limit of normal (ULN) values for ALA and PBG as 1.47 and 0.137 mmol/mol Cr, respectively. Relative to these ULN values, baseline urinary ALA and PBG levels in AHP patients were found to be 9.3‐ to 12‐fold, and 238‐ to 336‐fold higher, respectively. Results from this study can serve as a guide to assess the effectiveness of therapeutic interventions in lowering ALA and PBG.

## 1 INTRODUCTION

Acute hepatic porphyria (AHP) is a family of rare, serious, and potentially life‐threatening metabolic disorders caused by mutations in genes encoding enzymes involved in hepatic heme biosynthesis. These disorders include acute intermittent porphyria (AIP), hereditary coproporphyria (HCP), variegate porphyria (VP), and δ‐aminolevulinic acid (ALA) dehydratase (ALAD) deficient porphyria (ADP).^1^ AIP is the most common AHP subtype, occurring in ~80% of cases, due to a deficiency of the porphobilinogen deaminase enzyme. Patients with AHP may experience serious neurovisceral attacks, characterized by severe incapacitating pain in the abdomen, back, and limbs alongside a constellation of neurologic sequelae, including motor weakness, mental status changes, autonomic instability with hypertension, and seizures.^1^, ^2^, ^3^, ^4^, ^5^

AHP is characterized by induction of the heme synthesis pathway in the liver through the initial and rate‐limiting enzyme δ‐aminolevulinic acid synthase 1 (ALAS1) which results in the accumulation of heme intermediates, primarily ALA, and porphobilinogen (PBG). The preponderance of clinical evidence suggests ALA as the main contributor to AHP disease manifestations, including neurovisceral attacks,^5^, ^6^ but a contribution by PBG has not been ruled out.^7^ AHP patients have been shown to have substantially elevated ALA and PBG levels compared to healthy subjects in periods between attacks that further increase during porphyria attacks. In a natural history study in AHP patients experiencing recurrent attacks, mean ALA and PBG levels in AIP patients were elevated 8‐fold and 20‐fold above the upper limit of normal (ULN), respectively, in between attacks.^8^ Similarly, ALA and PBG levels have been reported to be elevated in a subpopulation of AHP patients who do not have acute neurovisceral attacks but are biochemically active, referred to as chronic high excreters (CHE), but these levels are in general 2‐fold lower than AHP patients with recurrent attacks.^7^, ^9^, ^10^, ^11^

Since elevated ALA and PBG are central to the pathogenesis of AHP, efforts to develop therapeutic treatments have been focused on reducing their levels toward those observed in the healthy population. Givosiran is a novel synthetic RNA interference (RNAi) therapeutic agent that specifically targets *ALAS1* messenger ribonucleic acid (mRNA) in the liver and has been recently approved under the tradename GIVLAARI for the treatment of adults with AHP.^12^ In phase I and phase 3 clinical trials in patients with AIP, givosiran‐mediated reduction of induced *ALAS1* mRNA was shown to reduce the accumulation of ALA and PBG consequently resulting in a significant decrease in the number of porphyria attacks.^11^, ^13^ Given the disease‐causal role of elevated ALA and PBG, an accurate determination of ALA and PBG levels in healthy subjects is key for relating the pharmacodynamic effect of clinical efficacy of therapeutics such as givosiran.

Previously, commercially available colorimetric assays have been used to measure ALA and PBG in human urine.^7^, ^9^, ^14^ However, these methods were developed to detect elevated urinary ALA and PBG in patients for diagnostic purposes. The assays were not intended for a different context of use such as quantifying ALA or PBG as a potential pharmacodynamic biomarker or efficacy endpoint in clinical studies, which requires better assay performance, especially specificity, accuracy, precision, and sensitivity.^15^ More specific, accurate, and reproducible liquid chromatography with tandem mass spectrometry (LC‐MS/MS) methods have therefore been developed for measuring urinary ALA and PBG.^16^, ^17^ Studies by Zhang et al and Benton et al quantified urinary ALA and PBG concentrations in healthy subjects using LC‐MS/MS methods; however, these studies did not provide the demographic characteristics of the study subjects or measures of data dispersion to enable the calculation of reference limits for ALA and PBG.

The current study quantified urinary ALA and PBG levels in healthy subjects using a new, highly sensitive, specific, and validated LC‐MS/MS method. The results were used to determine the distribution of ALA and PBG levels in healthy subjects and to establish their respective ULN values. Results from this study can serve as a guide to better understand the relationship between disease severity and baseline urinary ALA and PBG values and also to assess the effectiveness of therapeutic interventions in lowering urinary ALA and PBG.

## 2 METHODS

### 2.1 Urine samples from healthy subjects and AIP patients

Urine samples from 150 healthy subjects (subjects with no known significant health problems), were obtained from BioIVT (formerly Bioreclamation IVT), Westbury, New York, a commercial provider of biological samples. Samples were collected from consented donors under IRB approved protocols at BioIVT.

Additionally, baseline urine samples from untreated AIP patients with recurrent attacks (N = 17) and CHE subjects (N = 23) were obtained from a phase 1 clinical trial of givosiran (NCT02452372), and from EXPLORE (N = 86), a natural history study of AHP patients with recurrent attacks.^8^

### 2.2 Quantitation of ALA and PBG concentrations

Urine samples were stored under light protected conditions at −70°C until analyses. All steps during analyses were also conducted in light protected conditions. Briefly, ALA and PBG were quantified after derivatization with premixed 3 N HCl in N‐butanol solution (procured from Sigma Aldrich, St. Louis, Missouri). Both analytes and their isotopic internal standards were derivatized during the procedure. Samples were processed by a solid‐phase extraction procedure using 4% phosphoric acid and 2% formic acid, and analyzed using LC‐MS. The LC system was a Shimadzu Sil 30 system fitted with a 1.7 μM UPLC column maintained at 40°C. Eluent A was 0.1% formic acid in water and eluent B was a mixture of 50:50 v/v acetonitrile and methanol. Analytes were eluted using a stepwise gradient elution program. MS/MS (AB Sciex, Concord, Ontario, Canada) was carried out in positive ionization mode using electrospray ionization and selected reaction monitoring transitions of ALA (188.2 → 114.0) and PBG (322.2 → 222.0) were captured. Instrument response ratios for the standards were used to create a linear calibration curve for ALA and quadratic calibration curve for PBG using 1/x^2^ weighted least‐squares regression analysis. Please see Supporting Information for additional details of the LC‐MS method along with representative standard curves.

The assays were sensitive with lower limit of quantitation (LLOQ) of 10 ng/mL, selective and specific, accurate (% bias ≤15%), and precise (% CV ≤15%). Instrument response ratios for the standards were used to create a calibration curve from 10.0 to 3000 ng/mL.

Urinary creatinine concentrations were measured by the Jaffe method^18^ at Medpace Inc, Cincinnati, Ohio.

### 2.3 Statistical analysis

Urine concentrations of ALA and PBG were normalized to time‐matched urine creatinine (Cr) concentrations to correct for differences in urine volume and are presented as mmol/mol Cr [creatinine]. Normalization of urinary ALA and PBG levels with creatinine concentrations was considered appropriate because analysis of absolute ALA and PBG data (non‐normalized) showed a high correlation between ALA/PBG levels and creatinine levels (data not shown) indicating that differences in creatinine excretion (if any) between subjects did not affect their ALA/PBG results.

ALA and PBG levels were summarized using descriptive statistics. Concentrations below LLOQ or above the upper limit of quantitation (ULOQ) were excluded from the calculation of summary statistics.

A receiver operating characteristic (ROC) curve was constructed to evaluate the ability of different ULN thresholds to differentiate ALA and PBG levels in healthy subjects from AIP patients. Sensitivity and specificity of several ULN thresholds (median, 75th, 90th, 95th, and 99th percentile, and maximum value) for ALA and PBG were determined as follows:

$$\text{Sensitivity}=\text{True positive}/\left(\text{True positive}+\text{False negative}\right)$$

$$\text{Specificity}=\text{True negative}/\left(\text{True negative}+\text{False positive}\right)$$

where true/false positives and negatives were defined as below:

**Table:** 

Categorization based on ULN threshold	Actual disease status	
	Healthy	AIP patients
Healthy (≤ULN)	True negative	False negative
AIP patients (>ULN)	False positive	True positive

## 3 RESULTS

### 3.1 Healthy subject demographics

Urine samples from a total of 150 healthy subjects, including 90 females and 60 males, were included in this analysis. Subjects ranged from 19 to 64 years of age with an overall mean of 39.5 years, the majority were black (60%) followed by Caucasian (23%) (Table 1). Urine creatinine concentrations were in the normal range for majority of the subjects.

**TABLE 1:** Summary of demographic characteristic of healthy subjects

Parameter	Statistic	Value
Age (years)	Mean (SD)	39.5 (11.9)
	Median (min‐max)	38.0 (19.0‐64.0)
Sex, n (%)	Female	90 (60.0%)
	Male	60 (40.0%)
Race, n (%)	White/Caucasian	34 (22.7%)
	Black	90 (60.0%)
	Hispanic	23 (15.3%)
	Asian	3 (2.0%)
Creatinine concentration (mg/dL)	Mean (SD)	122 (74.3)
	Median (min‐max)	117 (6.60‐363)
	Mean (SD)—Females	109 (72.8)
	Mean (SD)—Males	143 (72.2)

*Note:* Creatinine concentrations can be converted to mol/L based on molecular weight of 113.12 g/mol.

Abbreviations: max, maximum; min, minimum.

### 3.2 Urine ALA and PBG levels in healthy subjects

**FIGURE 1:**
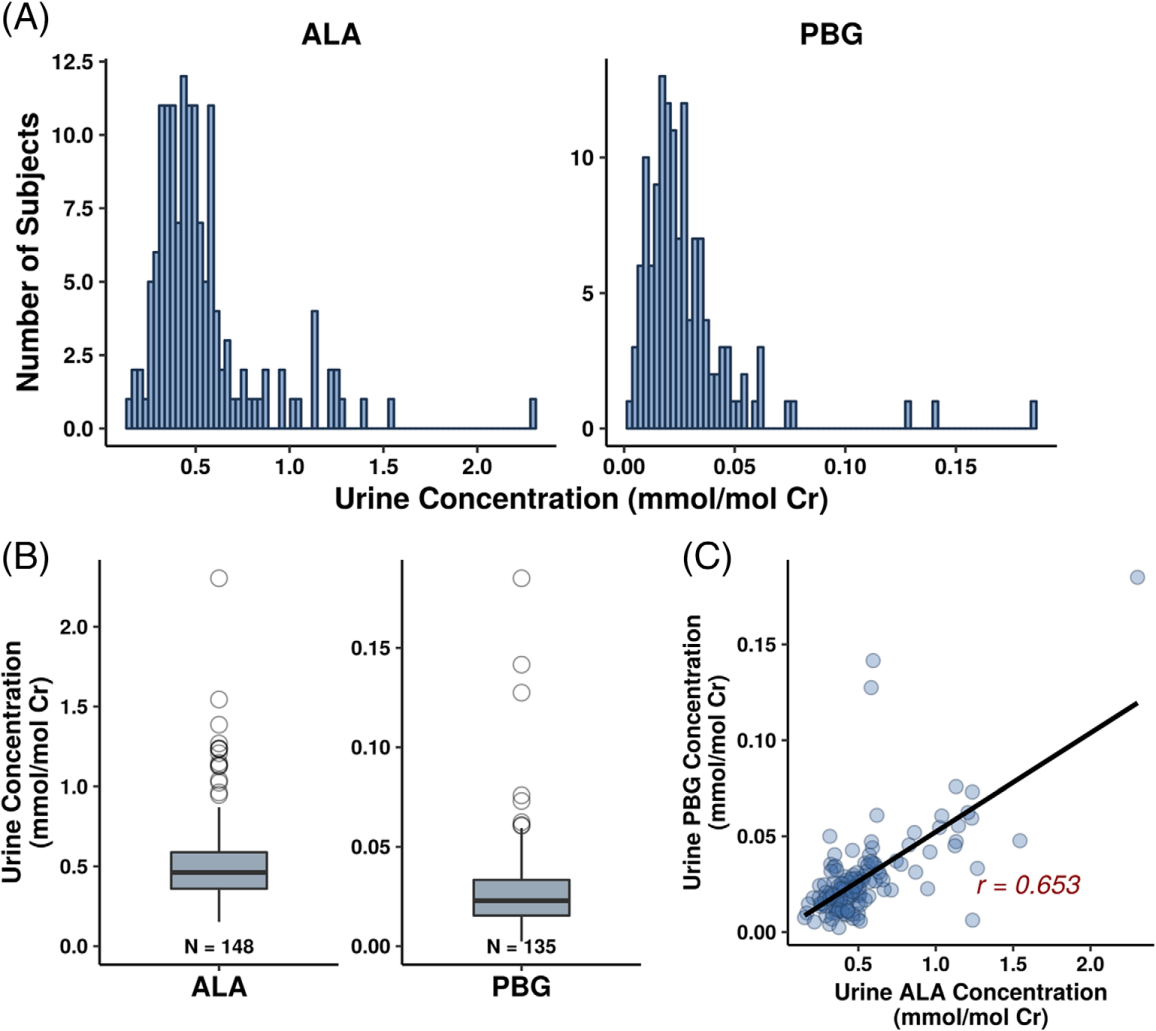
Distribution of urine ALA and PBG concentrations in healthy subjects (A, B), and correlation between urine ALA and PBG concentrations (C). ALA, aminolevulinic acid; Cr, creatinine; PBG, porphobilinogen

Urine ALA and PBG concentrations in healthy subjects were normally distributed (Figure 1A) with mean (±SD) values of 0.539 (±0.300) mmol/mol Cr and 0.0281 (±0.0238) mmol/mol Cr, respectively. Urine concentrations ranged from 0.152 to 2.30 mmol/mol Cr for ALA and from 0.00234 to 0.185 mmol/mol Cr for PBG (Table 2, Figure 1B). Two ALA values and two PBG values were greater than the 99th percentile, which were observed in three subjects: one female/black subject had the highest urine ALA and PBG concentrations at 2.30 and 0.185 mmol/mol Cr, respectively. One female/Caucasian subject's ALA level (1.54 mmol/mol Cr) and another female/Caucasian subject's PBG level (0.141 mmol/mol Cr) were slightly higher than the 99th percentile. Urine ALA and PBG concentrations were highly correlated with a Pearson correlation coefficient of 0.6527 (Figure 1C).

**TABLE 2:** Distribution of urine ALA and PBG levels in healthy subjects

	N	Mean (SD)	Median	Range (min, max)	5th percentile	99th percentile
ALA (mmol/mol Cr)^a^						
Overall	148	0.539 (0.300)	0.462	0.152–2.30	0.256	1.47
Females	89	0.613 (0.346)	0.513	0.152–2.30	0.237	1.64
Males	59	0.429 (0.162)	0.393	0.246‐1.24	0.258	1.02
PBG (mmol/mol Cr)^a^						
Overall	135	0.0281 (0.0238)	0.0229	0.00234–0.185	0.00706	0.137
Females	78	0.0349 (0.0288)	0.0280	0.00234–0.185	0.00832	0.152
Males	57	0.0190 (0.0082)	0.0185	0.00415‐0.0404	0.00669	0.0376

*Note:* Concentrations below LLOQ (N = 14 for PBG) or above ULOQ (N = 1 for ALA) were excluded from calculation of summary statistics. Additionally, ALA and PBG levels were not reported in 1 subject due to a measurement error. This subject was excluded.

Abbreviations: ALA, aminolevulinic acid; PBG, porphobilinogen; Cr, creatinine; LLOQ, lower limit of quantitation; max, maximum; min, minimum; ULOQ, upper limit of quantitation.

^a^ ALA and PBG levels are presented as mmol/mol Cr and were calculated by conversion of measure values in ng/mL to mmol/L based on molecular weight of 167.59 g/mol for ALA and 226.23 g/mol for PBG, and dividing by corresponding creatinine concentrations in mol/L.

### 3.3 Distribution of urine ALA and PBG levels by gender, race, and age

**FIGURE 2:**
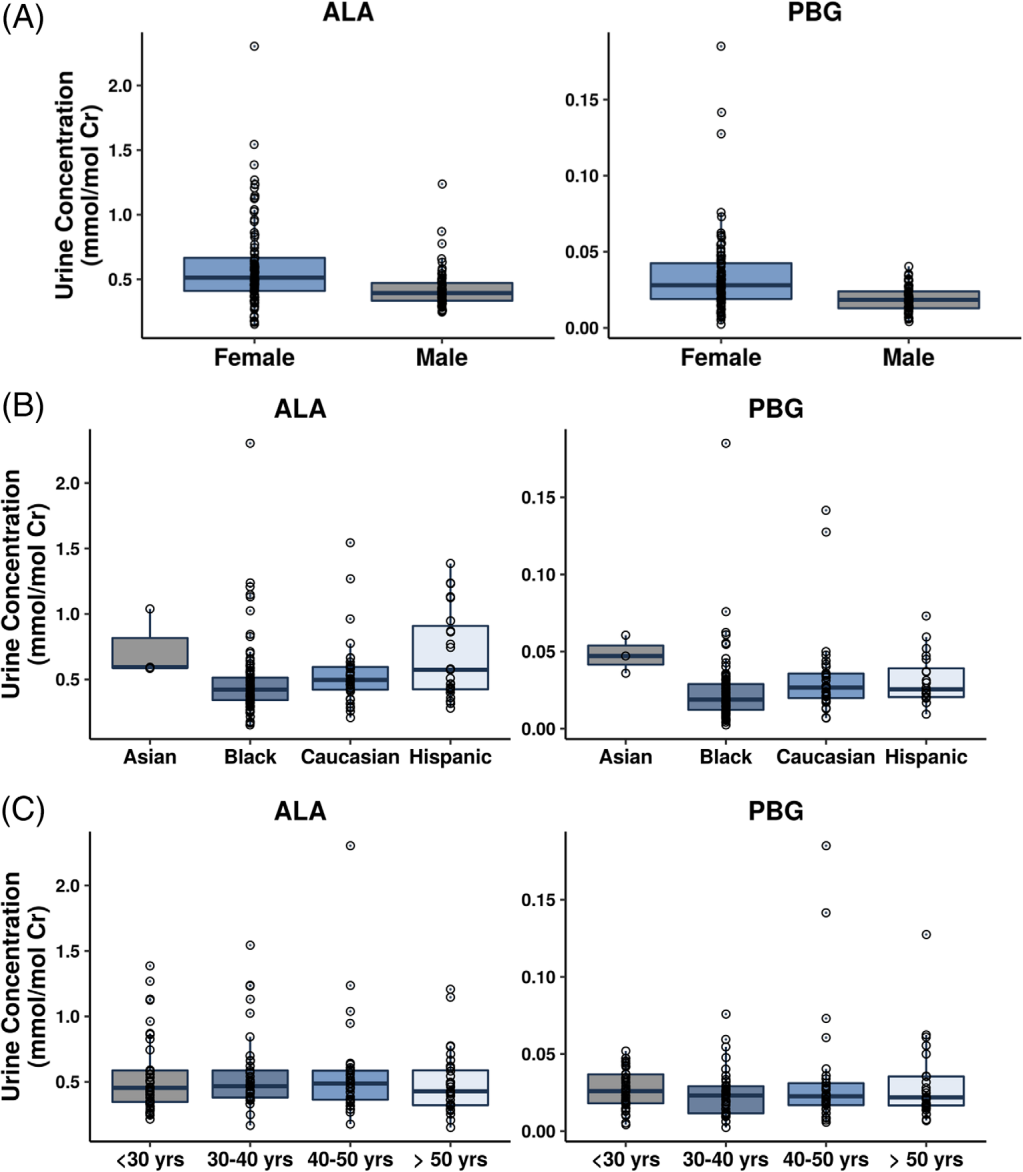
Distribution of urine ALA and PBG concentrations by gender (A), race (B), and age (C). ALA, aminolevulinic acid; Cr, creatinine; PBG, porphobilinogen

Mean ALA and PBG concentrations were numerically lower in male subjects than females (Table 2, Figure 2A). The mean (±SD) ALA concentration was 0.429 (±0.162) mmol/mol Cr in males and 0.613 (±0.346) mmol/mol Cr in females. Mean (±SD) PBG concentration was 0.0190 (±0.0082) mmol/mol Cr in males and 0.0349 (±0.0288) mmol/mol Cr in females.

There was considerable overlap in the range of ALA and PBG concentrations in black, Caucasian, and Hispanic subjects (Figure 2B). Asian subjects appeared to have higher median concentrations of both biomarkers; however, this may be an artifact of the small number (N = 3) of Asian subjects in the dataset. One black subject had the highest observed value for ALA (2.30 mmol/mol Cr) and PBG (0.185 mmol/mol Cr).

ALA and PBG values by age were evaluated by grouping subjects into four age brackets, 19‐30 years, 30‐40 years, 41‐50 years, 50‐64 years. There was no difference in the distribution of urine ALA and PBG concentrations in subjects across the age range of 19 to 64 years (Figure 2C). Mean ALA concentrations across the 4 age brackets were within 15% of each other, while mean PBG concentrations were within 30% across the different age brackets.

### 3.4 Determination of upper limit of normal for urine ALA and PBG

Urine ALA and PBG concentrations were normally distributed (Figure 1A) with no clear outliers and 98% of subjects falling within 3 SDs from the mean (ALA and PBG concentrations >3 × SDs from the mean were observed in only 2 and 3 subjects, respectively). Therefore, the ULN for ALA and PBG were set to the 99th percentile of the data, at 1.47 and 0.137 mmol/mol Cr, respectively (Table 2). Given the overlapping range of urine ALA and PBG concentrations across gender, race, and age categories, a single ULN value was considered sufficient across all subjects.

### 3.5 Sensitivity and specificity of the upper limit of normal in separating healthy subjects from AIP patients

**FIGURE 3:**
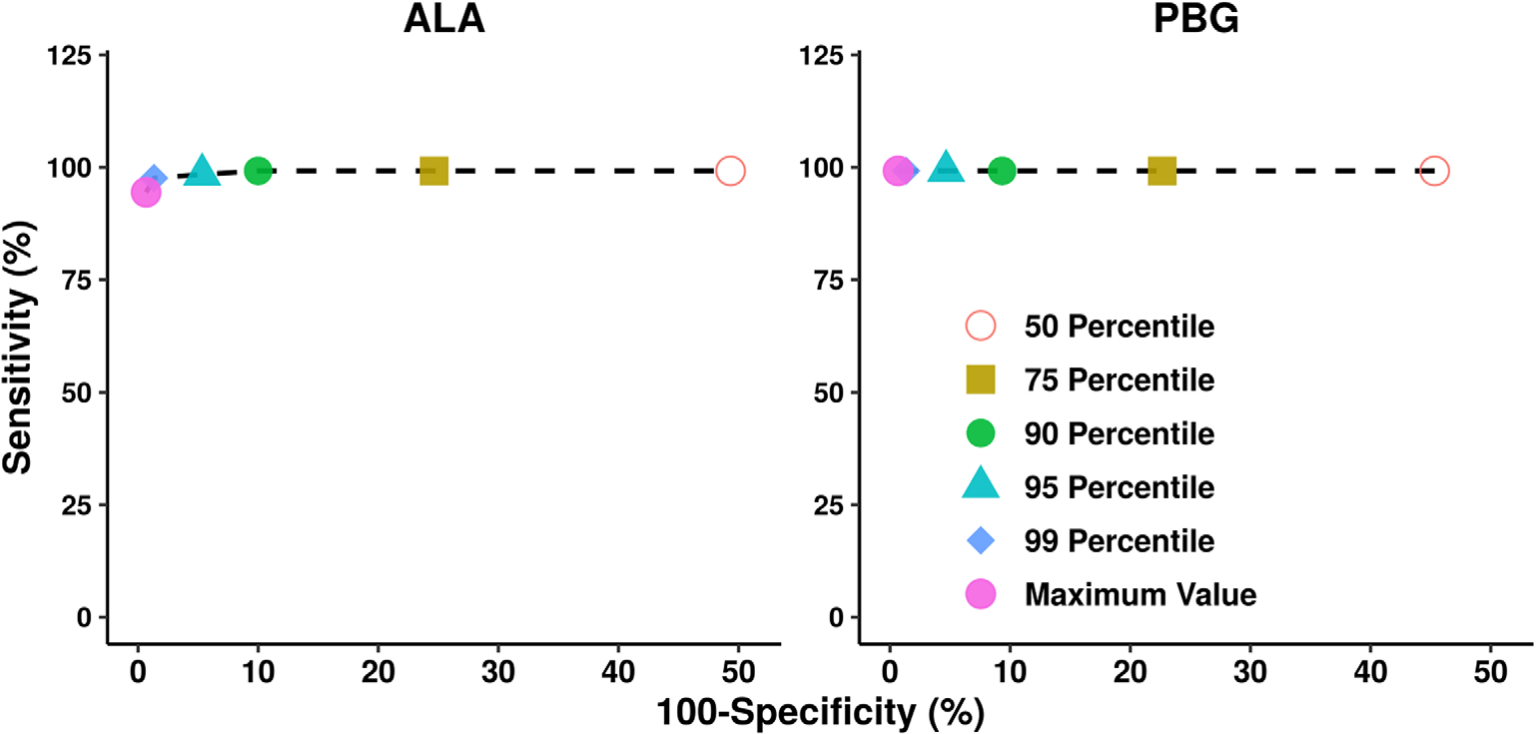
Receiver operating characteristic (ROC) curves for ALA and PBG. ROC curves were constructed to evaluate the ability of different ULN thresholds (median, 75th, 90th, 95th, and 99th percentile, and maximum value) in differentiating ALA and PBG levels in healthy subjects from AIP patients. Baseline ALA and PBG levels from AIP patients in the phase 1 and EXPLORE studies were used. AIP, acute intermittent porphyria; ALA, aminolevulinic acid; PBG, porphobilinogen; ULN, upper limit of normal

ROC curves (Figure 3) were constructed using baseline urine ALA and PBG concentrations in AIP patients in the phase 1 and EXPLORE studies. The ROC curves indicate that use of the 99th percentile of urine ALA and PBG levels as the ULN results in a sensitivity and specificity of 98% in differentiating healthy subjects from AIP patients.

### 3.6 Comparison of urine ALA and PBG concentrations in healthy subjects and AIP patients

**FIGURE 4:**
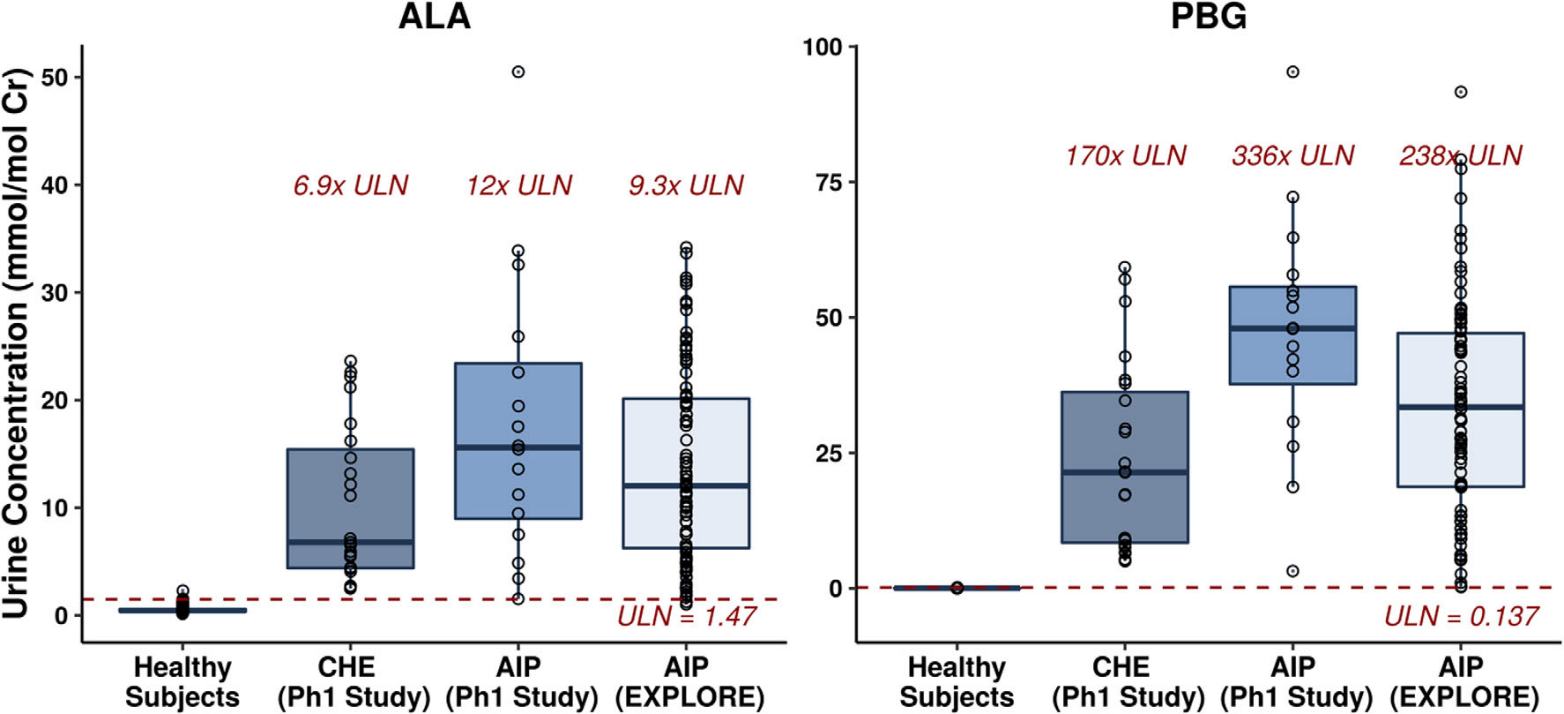
Comparison of urine ALA and PBG levels between healthy subjects and AIP patients. Baseline ALA and PBG levels from AIP patients in the phase 1 and EXPLORE studies were used for comparison to healthy subjects. AIP, acute intermittent porphyria; ALA, aminolevulinic acid; CHE, chronic high excreters; Cr, creatinine; PBG, porphobilinogen; Ph, phase; ULN, upper limit of normal

In the phase 1 study, mean baseline ALA and PBG concentrations in CHE subjects were 6.9‐fold and 170‐fold higher than the respective ULN values. Mean baseline ALA and PBG concentrations in AIP patients in the study were 12‐fold and 336‐fold higher than the respective ULN values. Similarly, mean baseline levels of ALA and PBG in AIP patients from EXPLORE were 9.3‐fold and 238‐fold above the ULN. There was minimum overlap in the range of ALA and PBG concentrations between AIP patients from the phase 1, and EXPLORE studies and healthy subjects (Figure 4, Table 3).

**TABLE 3:** Comparison of urine ALA and PBG levels in healthy subjects vs AIP patients

	N	Mean (SD)	Median	Range (min, max)	Ratio (mean/ULN^a^)
ALA (mmol/mol Cr)					
Healthy subjects	148	0.539 (0.300)	0.462	0.152‐2.30	NA
CHE subjects	23	10.3 (7.24)	6.8	2.5‐23.6	6.9
AIP (phase 1 study)	16	17.8 (13.0)	15.6	1.54‐50.5	12.0
AIP (EXPLORE)	86	14.0 (8.87)	12	1.03‐34.2	9.3
PBG (mmol/mol Cr)					
Healthy subjects	135	0.0281 (0.023)	0.0229	0.00234‐0.185	NA
CHE subjects	23	23.8 (17.5)	21.4	5.03‐59.3	170
AIP (phase 1 study)	16	47.0 (21.6)	47.9	3.20‐95.3	336
AIP (EXPLORE)	86	33.3 (20.0)	33.5	0.242‐91.6	238

Abbreviations: AIP, acute intermittent porphyria; ALA, aminolevulinic acid; CHE, chronic high excreters; Cr, creatinine; max, maximum; min, minimum; NA, not applicable; PBG, porphobilinogen; ULN, upper limit of normal.

^a^ ULN = 1.47 mmol/mol Cr for ALA and 0.137 mmol/mol Cr for PBG.

## 4 DISCUSSION

Elevated levels of ALA and PBG are used to diagnose AHP patients and to characterize periods of disease exacerbation, such as during neurovisceral attacks. ALA and PBG levels in AIP patients have been reported to be 16‐fold and 50‐fold higher than healthy subjects using colorimetric assays.^19^ However, only few studies have reported the distribution and intersubject variability of ALA and PBG levels in healthy subjects. This study quantified urinary ALA and PBG levels in 150 healthy subjects using a validated LC‐MS/MS assay and used the distribution of ALA and PBG values to establish ULN values in healthy subjects.

The observed range of urine concentrations of ALA (0.152‐2.30 mmol/mol Cr) and PBG (0.00234‐0.185 mmol/mol Cr, Table 2) were generally consistent with the ranges reported in literature (0.09‐3.0 mmol/mol Cr for ALA and 0.0‐1.1 mmol/mol Cr for PBG).^16^, ^17^ However, median ALA and PBG levels in the current study were considerably lower than previously reported values using LC‐MS/MS and colorimetric assays.^14^, ^16^, ^17^, ^20^ This difference could be due to the higher sensitivity and specificity of the current LC‐MS/MS method. The LLOQ in this study was lower (10 ng/mL for ALA and PBG) compared to most published LC‐MS/MS methods (8‐500 ng/mL for ALA, and 11‐450 ng/mL for PBG)^16^, ^17^ and colorimetric assays (400‐450 ng/mL for ALA, and 320‐1340 ng/mL for PBG).^14^, ^20^ Additionally, the LC‐MS/MS method used in this study has better selectivity compared to colorimetric methods that are generally prone to overestimation of results due to interference of test reagents or other components of biological matrices such as blood and urine.^21^, ^22^, ^23^

Contrary to previous reports in the literature where the ratio of PBG/ALA in healthy subjects was 0.32, in this study the PBG/ALA ratio was found to be considerably lower at ~0.05. This is likely due to the substantially lower PBG levels observed in the current study.^20^ Floderus et al reported mean PBG levels of 0.54 mmol/mol Cr based on samples from five healthy subjects, whereas we report a mean of 0.0281 mmol/mol Cr from 150 healthy subjects. As mentioned earlier, the lower PBG levels in the current study could be due to the higher specificity of the assay used for detection in the current study. This is corroborated by the PBG/ALA ratio of 0.07 reported by Zhang et al who quantified ALA and PBG using an LC‐MS/MS assay with similar LLOQ as that in the current study.

Since AHP predominantly impacts young women (~80%),^8^, ^9^ we evaluated the influence of gender and age on the distribution of urinary ALA and PBG levels. Females appeared to have slightly higher mean ALA and PBG levels compared to male subjects. However, there was considerable overlap in the range of urinary ALA and PBG concentrations between males and females (Figure 2), and therefore, separate ULN values were not considered necessary based on gender. Similarly, the range of urine ALA and PBG concentrations was comparable across age categories (Figure 2). These results are corroborated by the work of Dixon et al who demonstrated a lack of correlation between age and excretion of ALA and PBG in urine of AHP patients.^24^

Based on the overall distribution of ALA and PBG, the 99th percentile values of 1.47 and 0.137 mmol/mol Cr were defined as the ULN for urinary ALA and PBG, respectively. The ROC analysis confirmed that the threshold of 99th percentile for ULN provided near maximum sensitivity and specificity for differentiating AHP patients and healthy subjects (Figure 3). This was also confirmed by the comparison of urinary ALA and PBG levels in healthy subjects from the current study to that in AIP patients and CHE subjects in the phase I clinical trial of givosiran as well as the EXPLORE natural history study. There was minimal overlap in ALA and PBG levels between healthy subjects and AIP patients or CHE subjects; only 2/102 (2%) AIP patients had urinary ALA levels slightly lower than the ULN (values of 1.03 and 1.27 mmol/mol Cr) (Figure 4). Thus, the established ULN provides an adequate threshold to differentiate the ALA and PBG levels of healthy subjects from that of CHE subjects or AIP patients.

Comparison of mean urinary ALA and PBG concentrations in CHE subjects and AIP patients to the established ULN values in healthy subjects indicate that ALA and PBG concentrations in AIP patients are 9‐ to 12‐fold and 238‐ to 336‐fold over the ULN, respectively (Table 3). AIP is the most common subtype of AHP with other subtypes such as VP, HCP very rare. Given the low incidence of the other subtypes, the majority of the clinical experience in EXPLORE and Study 001 is from AIP patients. Even so, we believe that the ULN values established here are applicable to the entire AHP population. This is based on data from EXPLORE where even though baseline ALA and PBG levels in patients with VP and HCP were lower than that in AIP patients, they were still 2.4‐fold and 11.4‐fold above the ULN.^8^ Furthermore, the results in this manuscript demonstrate that the ULN values are able to differentiate even CHE subjects who are known to have relatively low ALA and PBG levels^19^compared to other AHP patients, thereby corroborating the use of these ULN values for the entire AHP population. Thus, the ULN values established in this study may serve to facilitate diagnosis of AHP and to evaluate the pharmacodynamic effect of current and future therapies in AHP.

## CONFLICT OF INTEREST

Sagar Agarwal, Bahru Habtemarium, Yuanxin Xu, Amy R. Simon, Jae B. Kim, and Gabriel J. Robbie declare that they have no conflict of interest.

## AUTHOR CONTRIBUTIONS

Sagar Agarwal: designed research, conducted analysis, wrote manuscript; Bahru Habtemarium: designed research, reviewed manuscript; Yuanxin Xu: designed research, conducted bianalysis; Amy R. Simon: designed research, reviewed manuscript; Jae B. Kim: designed research, reviewed manuscript; Gabriel J. Robbie: designed research, wrote manuscript.

## INFORMED CONSENT

All procedures followed were in accordance with the ethical standards of the responsible committee on human experimentation (institutional and national) and with the Helsinki Declaration of 1975, as revised in 2000 (5). Informed consent was obtained from all donors who contributed samples for the study.

## Supporting information


**Data S1** Supporting information
