# Supplementary material for: Normal reference ranges for urinary δ‐aminolevulinic acid and porphobilinogen levels
Source: JIMD Rep. 2020 Oct 1;57(1):85–93. doi: 10.1002/jmd2.12173 (PMC7802627; doi:10.1002/jmd2.12173)

**Quantitation of ALA and PBG Concentrations**

Urine samples were stored under light protected conditions at -70°C until analyses. All steps during analyses were also conducted in light protected conditions. Briefly, ALA and PBG quantified after derivatization with premixed 3N HCl in N-butanol solution (procured from Sigma Aldrich). Both analytes and their isotopic internal standards were derivatized during the procedure. Samples were processed by a solid-phase extraction procedure using 4% phosphoric acid and 2% formic acid, and analyzed using LC-MS. The LC system was a Shimadzu Sil 30 system fitted with a 1.7 µM UPLC column maintained at 40°C. Eluent A was 0.1% formic acid in water and eluent B was a mixture of 50:50 v/v acetonitrile and methanol. Analytes were eluted using a stepwise gradient elution program as follows –

| **Time (mins)** | **Eluent A** | **Eluent B** |
| --- | --- | --- |
| 0.01 | 90% | 10% |
| 2.5 | 70% | 30% |
| 3.5 | 20% | 80% |
| 4.7 | 20% | 80% |
| 4.8 | 90% | 10% |

MS/MS (AB Sciex, Concord, Ontario, Canada) was carried out in positive ionization mode using electrospray ionization and selected reaction monitoring transitions of ALA (188.2 → 114.0) and PBG (322.2 → 222.0) were captured.

Instrument response ratios for the standards were used to create a linear calibration curve for ALA and quadratic calibration curve for PBG using 1/x2 weighted least-squares regression analysis. Representative standard curves are presented below –

**Representative Calibration Curve for ALA in Human Urine**


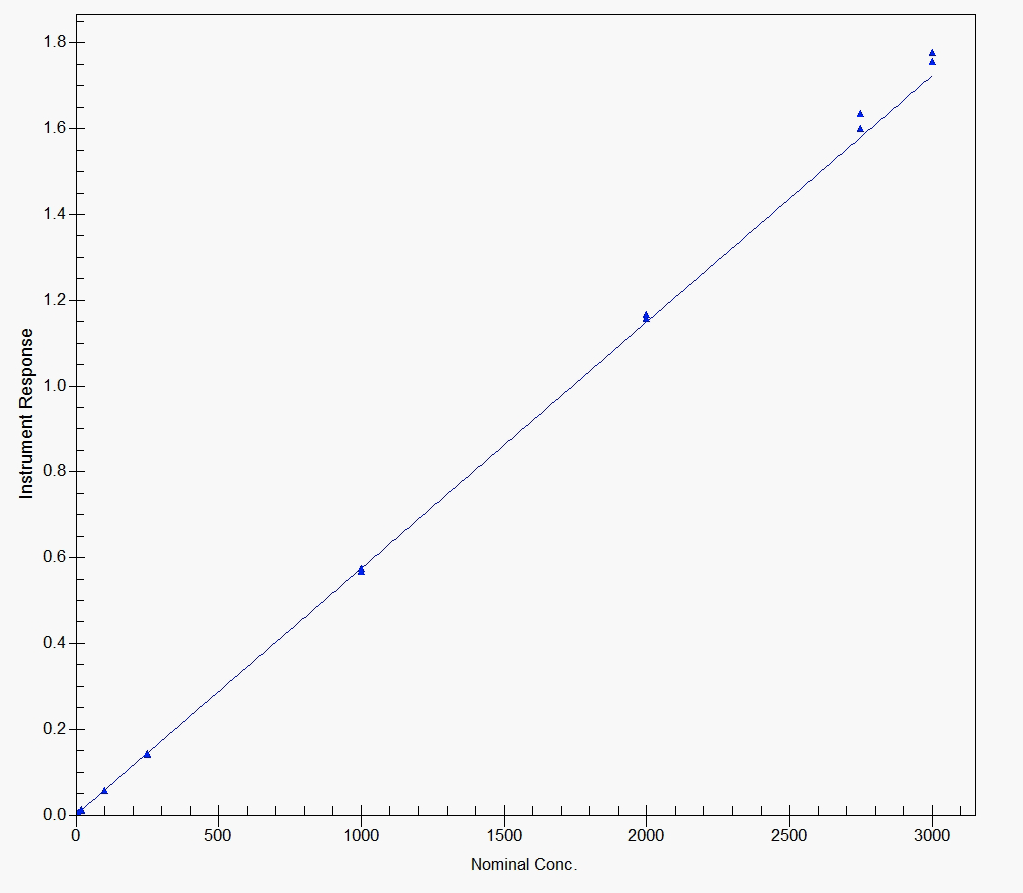


**Representative Calibration Curve for PBG in Human Urine**


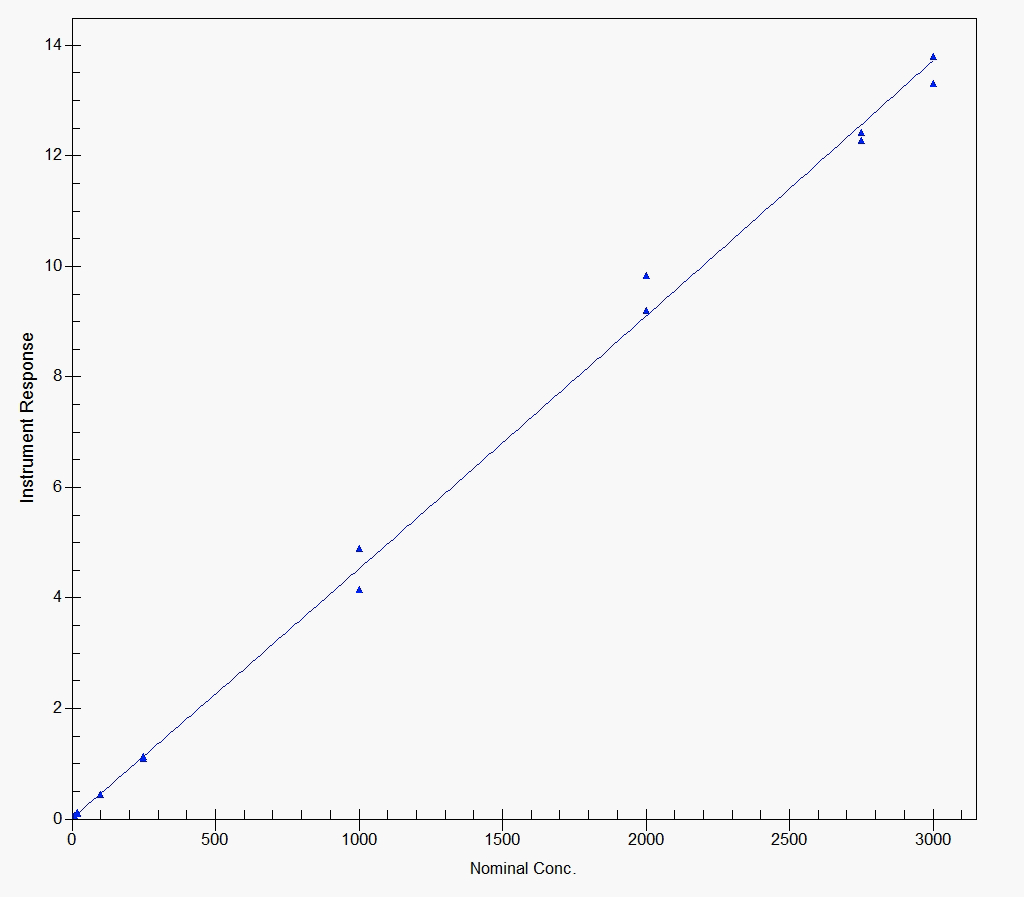

Supplement: Supplementary file 1 — Data S1 Supporting information [file JMD2-57-85-s001.docx]
